# Contributions of extracellular-signal regulated kinase 1/2 activity to the memory trace

**DOI:** 10.3389/fnmol.2022.988790

**Published:** 2022-10-05

**Authors:** Santiago Ojea Ramos, Mariana Feld, María Sol Fustiñana

**Affiliations:** ^1^Instituto de Fisiología, Biología Molecular y Neurociencias, Universidad de Buenos Aires-Consejo Nacional de Investigaciones Científicas y Técnicas, Buenos Aires, Argentina; ^2^Friedrich Miescher Institute for Biomedical Research, Basel, Switzerland

**Keywords:** long term memory (LTM), consolidation, engram, subcellular localization, long term potentiation (LTP), temporal integration, spacing effect, isoforms

## Abstract

The ability to learn from experience and consequently adapt our behavior is one of the most fundamental capacities enabled by complex and plastic nervous systems. Next to cellular and systems-level changes, learning and memory formation crucially depends on molecular signaling mechanisms. In particular, the extracellular-signal regulated kinase 1/2 (ERK), historically studied in the context of tumor growth and proliferation, has been shown to affect synaptic transmission, regulation of neuronal gene expression and protein synthesis leading to structural synaptic changes. However, to what extent the effects of ERK are specifically related to memory formation and stabilization, or merely the result of general neuronal activation, remains unknown. Here, we review the signals leading to ERK activation in the nervous system, the subcellular ERK targets associated with learning-related plasticity, and how neurons with activated ERK signaling may contribute to the formation of the memory trace.

## Introduction

One of the major questions in neuroscience is how the brain integrates the different external stimuli to generate an internal representation that can be evoked at a particular time point. In other words, how are memories formed in the brain? A strong body of work has described how different molecular signaling pathways shape learning-associated synaptic plasticity mechanisms. More than two decades ago the extracellular-signal regulated kinases 1 and 2 (ERK) subfamily of mitogen-activated protein kinases (MAPKs) was proposed as a critical player in synaptic and neuronal plasticity (Martin et al., 1997; Atkins et al., 1998). Its role in these processes has been shown in different species, brain areas, types of synapses and even synaptic compartments. Moreover, dysregulation of ERK signaling has been linked to learning disorders (Costa et al., 2002; Kyosseva, 2004; Sanderson et al., 2016) and addiction (Lu et al., 2006; Sun et al., 2016).

Although increasing efforts have been made to elucidate the molecular mechanisms underlying memory formation, it is still unclear how the different elements contribute to the formation of the memory trace. Here, we review relevant work that settles ERK as an essential and integrative element into the complex memory theoretical framework. Understanding the molecular basis of memory formation may contribute to the development of new therapies for brain disorders.

## Extracellular-signal regulated kinase/mitogen-activated protein kinase pathway

**FIGURE 1:**
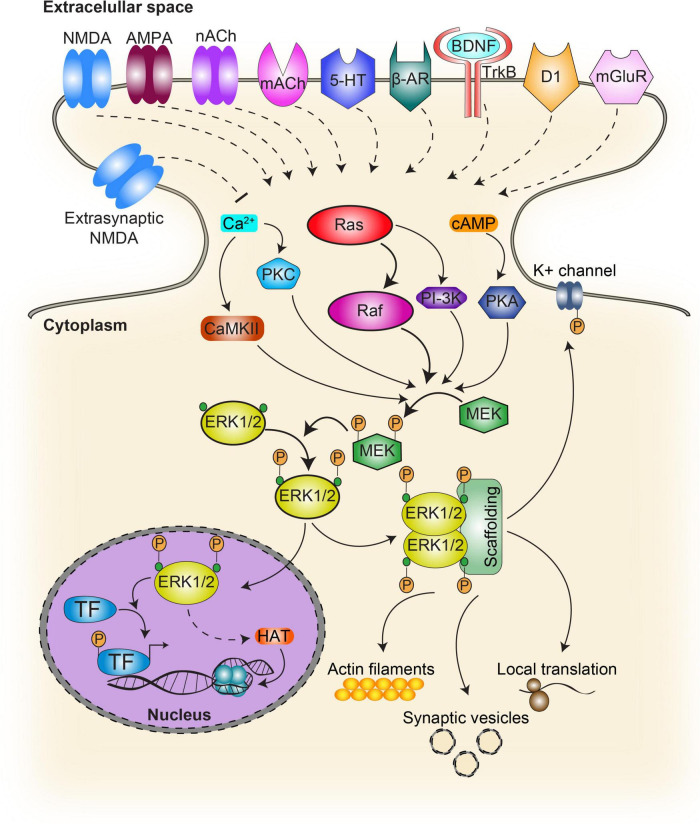
Schematic diagram of the activation pathways and targets of ERK. In the classical ERK cascade, activation of the receptor upon ligand binding results in the recruitment of the Ras family protein activating Raf. This step initiates the sequential phosphorylation of MEK which in turn activates ERK. Phosphorylated ERK targets cytosolic as well as nuclear substrates. Several other signaling pathways contribute to ERK activation. G-protein coupled receptors prompt the intracellular production of cAMP and calcium, while calcium can also increase intracellularly through ionotropic receptors. While cAMP contributes to ERK activation through PKA, calcium does it by molecules such as PKC and CaMKII. NMDA, NMDA receptors; nACh, nicotinic acetylcholine receptor; mACh, muscarinic acetylcholine receptor; 5-HT, serotonin receptor; β-AR, beta adrenergic receptor; BDNF, brain-derived neurotrophic factor; TrkB, tropomyosin receptor kinase B, also known as tyrosine receptor kinase B; D1, type 1 dopamine receptor; TF, transcription factors such as CREB and Elk1; HAT, histone acetyltransferase. Continuous lines indicate direct action while dash lines indicate indirect action.

Extracellular-signal regulated kinase/mitogen-activated protein kinases are known to couple a wide range of extracellular signals to major cellular programs such as proliferation, differentiation and apoptosis in a variety of species and tissues (Cobb et al., 1994; Robbins et al., 1994). They were the first kinases among the big family of MAPKs to be discovered (Boulton and Cobb, 1991) and consequently one of the most studied regarding mechanisms of brain plasticity, learning, and memory (for a review see Sweatt, 2004). Their activation mechanisms and functions have been described elsewhere (Davis, 1995; Thomas and Huganir, 2004; Yoon and Seger, 2006; Casar and Crespo, 2016; Miningou and Blackwell, 2020), so we are not going to get into further detail. Briefly, ERKs are Serine/Threonine (Ser/Thr) protein kinases from the highly-conserved family of the MAPKs which become activated by extracellular signals operating mainly, though not exclusively, through receptor tyrosine kinases (RTKs). In the nervous system, RTKs are typically activated by growth factors or neurotrophins which activate Ras (a superfamily of small G proteins) acting through the Grb2 adaptor protein and SOS (a guanyl nucleotide exchange factor, GEF). Ras superfamily depend on GTPase activating proteins (GAPs) to accelerate GTP hydrolysis, and GEFs to switch from the inactive (GDP bound) to the active (GTP bound) form. The active protein subsequently triggers activation of a general cascade motif of three sequential kinases: a MAPKKK from the Raf family (mostly Raf-1 and B-Raf in the brain); a MAPKK also called MEK (MAPK/ERK Kinase) and the MAPK effector, ERK for the purpose of this review (Thomas and Huganir, 2004; Figure 1). However, active members of the Ras superfamily can trigger other pathways as well (Stornetta and Zhu, 2011; Miningou and Blackwell, 2020). ERK becomes active upon dual phosphorylation specifically at Thr and Tyrosine (Tyr), inserted in a Thr-X-Tyr (TEY) motif, by MEK, although MEK-independent activation has been seldom reported (Aksamitiene et al., 2010; Simard et al., 2015). Dual phosphorylation is both necessary and sufficient for ERK activation (Canagarajah et al., 1997). On the contrary, dephosphorylation of either Thr, Tyr or both residues by Tyr-phosphatases, Ser/Thr-phosphatases or MAP kinase phosphatases (MKPs), a subgroup of dual-specificity phosphatases (DUSPs), returns MAPKs to the inactive state (Caunt and Keyse, 2013).

Extracellular-signal regulated kinase activation kinetics has also shown to be plastic and to influence cellular fate. While stimulation of Rat pheochromocytoma PC-12 cells with epidermal growth factor (EGF) induced transient Ras-dependent ERK activation leading to cell proliferation, nerve growth factor (NGF) incubation led to sustained ERK activation for hours, neurite outgrowth and cell differentiation into neurons (Traverse et al., 1992). However, this output also relied on cell-specific expression of ERK pathway-activating components such as B-Raf (Vossler et al., 1997). Thus, although the central motif in ERK activation pathway is conserved among species and cellular subtypes (e.g. Raf becomes activated and subsequently activates MEK, which then activates ERK), differences have been described in terms of either ERK negative feedback loops towards Raf or their upstream activation pathways (Miningou and Blackwell, 2020), which can account for different cellular outputs.

Another key to ERK-activation timing and substrate specificity also relies on scaffolding components as well as other molecular components recruitment such as kinases or phosphatases (Vaudry et al., 2002). ERK scaffold proteins include KSR1/2, IQGAP1 (IQ Motif Containing GTPase Activating Protein 1), MP1, MORG1, arrestin 1/2, Sef, MEKK1, and paxillin (Roskoski, 2012). However most of them have not been associated with plasticity and memory mechanisms yet. In the nervous system, ERK/MAPKs display a wide range of activation mechanisms, including those acting either *via* Ca^2 +^ signaling (e.g. glutamate and nicotine) or else *via* GPCRs coupled to PKA/PKC (e.g. dopamine, glutamate, opioids, and cannabinoids) (Sweatt, 2004; Thomas and Huganir, 2004), which may stand for the remarkable heterogeneity of cellular responses involved in memory and plasticity (Figure 1).

Several single neurotransmitters are able to activate the ERK pathway, some will be addressed in the following sections. Interestingly, ERK phosphorylation can be enhanced by activation of more than one neurotransmitter-receptor pathway (Girault et al., 2007). This phenomena has been observed in the hippocampus by the co-activation of β-adrenergic (β-AR) and cholinergic receptors (Watabe et al., 2000) and by the convergence of N-methyl-D-aspartate (NMDA) and dopamine receptors (Kaphzan et al., 2006). This dopamine and glutamate convergence was also observed in the striatum, linked to the mechanisms underlying drugs of abuse (Valjent et al., 2005; Voulalas, 2005). Taken together, this evidence suggests that ERK could be acting as a coincidence detector (Adams and Sweatt, 2002), although it still remains unknown if this mechanism is necessary for memory formation.

## Upon extracellular-signal regulated kinase activation

Once ERK is phosphorylated in the cytosol, it can translocate into the nucleus and interact with nuclear substrates to induce specific programs of gene expression (Davis, 1995; Klein et al., 2013). Although MAPKs were shown to exert their function at cytoplasmic as well as nuclear cellular compartments (Figure 1), the latter is probably the most widely studied and several functions have been described including regulation of transcription, DNA replication, chromatin remodeling, and miRNA synthesis. Regulatory components, such as scaffold proteins and dimerization were shown to take part in this pathway’s complex regulation by defining frequency, amplitude and intensity of the signal allowing for a wide range of biological outcomes (Herrero and Crespo, 2021). Several reports suggest that ERK nuclear localization depends, among others, on ERK expression levels such that overexpression increases nuclear translocation probability by passive diffusion (Fukuda et al., 1997), and phosphorylation by casein kinase 2 (CK2) that enhances ERK interaction with a nuclear import protein (importin 7) (Chuderland et al., 2008). In contrast, cytoplasmic localization depends on anchors expression levels (e.g. MEK; Fukuda et al., 1997), MAP kinase phosphatase 3 (MKP-3), which can dephosphorylate and consequently inactivate ERK (Brunet et al., 1999) or scaffolds such as the actin cytoskeleton-interacting protein IQGAP1, which mediates ERK binding to actin filaments (Vetterkind et al., 2013); as well as NR2A-induced ERK activation regulating dendritic spine density in key brain areas involved in cognition (Gao et al., 2011).

N-methyl-D-aspartate receptor (NMDAR) subunit composition is another contributing factor in the regulation of ERK activation and localization. Subunit-specific antagonization has shown differential responses in terms of nuclear propagation of ERK signals, leading to upregulation of the downstream nuclear targets pMSK1 and the immediate early gene product c-Fos, or membrane retention of phosphorylated ERK resulting in a lack of activation of these targets, which might underlie their specific roles in the formation of contextual and trace fear memory (Gao et al., 2010). It has been suggested that preferential coupling of NR2B to SynGAP could explain the subtype-specific function of NR2B-NMDARs in inhibition of Ras-ERK, removal of synaptic AMPA receptors (AMPARs), and weakening of synaptic transmission (Kim et al., 2005). Noteworthily, postsynaptic scaffolding protein PSD-95 was shown to regulate postsynaptic Ras activation, probably involving its interaction with the GTPase activating protein synGAP (Komiyama et al., 2002). NR2B-induced coupling NMDARs to ERK activation was shown to be mediated in the hippocampus by RasGRF1, a Ca^2 +^/calmodulin-dependent Ras-guanine-nucleotide-releasing factor (Krapivinsky et al., 2003), which impaired specifically long-term amygdala- (Brambilla et al., 1997) and hippocampus-related memory (Giese et al., 2001).

Interestingly, in addition to their synaptic location, NMDARs can also be found in the extrasynaptic membrane space (Tovar and Westbrook, 2002; Petralia et al., 2010) and are capable of bidirectional ERK signaling modulation depending on membrane structure localization. Thus, stimulation of synaptic NMDARs was shown to lead to ERK phosphorylation (Ivanov et al., 2006), whereas extrasynaptic NMDARs activation, which contributes to excitotoxicity, promotes dephosphorylation or no activation of ERK (Ivanov et al., 2006; Léveillé et al., 2008).

Among the myriad of ERK nuclear substrates, the transcription factor cAMP response element-binding protein (CREB) is selectively activated in neurons that are recruited into the memory trace (Han et al., 2007). In addition, the ternary complex factor Elk-1 is a key transcriptional regulator of serum response element (SRE)-driven gene expression which regulates immediate early gene (IEG) promoters such as junB and zif268 (also called early growth response gene-1, egr-1). Long-term potentiation (LTP) induction in the rat hippocampus triggers hyperphosphorylation of CREB and Elk-1 by ERK, leading to Zif268 expression (Roberson et al., 1999; Davis et al., 2000). Likewise, Elk-1 is phosphorylated in the insular cortex during the formation of aversive conditioning (Berman et al., 1998). Similarly, electrical NMDA-dependent long-term depression (LTD) induction in the hippocampal CA1 area induced a robust increase in nuclear ERK and Elk-1 phosphorylation which was completely blocked by the MEK inhibitor SL327 (Thiels et al., 2002). In addition, Elk-1 can promote the recruitment of the Srb mediator and coactivators, including CREB binding protein (CBP) and p300, a CBP-related protein (Besnard et al., 2011). Both, CBP and p300 have intrinsic histone acetyltransferase (HAT) activity and can associate with HATs that acetylate core histones, relieving repression of transcription through chromatin decompaction. Histone acetylation has been shown to be a hallmark of memory strength (Federman et al., 2012) and persistence (Federman et al., 2013), and has been proposed as an evolutionary conserved feature of memories (Federman et al., 2014).

Numerous regulatory functions of ERK have been described in the cytosol (Figure 1), such as synaptic vesicle trafficking (Earnest et al., 1996), increased probability of vesicle fusion *via* synapsin I (Vara et al., 2009; Giachello et al., 2010), local translation initiation (Gong and Tang, 2006; Leal et al., 2013), modulation of potassium currents through Kv4.2 channels (Yuan et al., 2002; Schrader et al., 2006) and the activation of other cell signaling pathways such as NF-kappaB (Jiang et al., 2004) which have been shown to be relevant for memory processes (Romano et al., 2006; Salles et al., 2014; de la Fuente et al., 2015). In addition, arrestins facilitate ERK activation by G protein-coupled receptor, but inhibit ERK-dependent transcription by retaining phosphorylated ERK (pERK) in the cytosol (Tohgo et al., 2002). Furthermore, extra-nuclear activation of ERK has been pointed out as a relevant part of learning and memory encoding in crabs and mice, two phylogenetically distant animal models (Feld et al., 2005; Krawczyk et al., 2015, 2016). It has been proposed that after phosphorylation, the dimerization of this kinase would be critical for the activation of cytosolic targets, allowing their union (Casar et al., 2008). These signaling pathways through post-translational modifications involved in plasticity and memory could be regulating signaling processes in different subcellular compartments such as dendrites.

## Extracellular-signal regulated kinase in plasticity, learning, and memory

In 1921, the term “engram” was coined by Dr. Semon to refer to the physical substrate of memory (Semon, 1921). Since then, many efforts have been focused on understanding how the engram is assembled (for reviews see Josselyn et al., 2015; Josselyn and Tonegawa, 2020). Likewise, synaptic plasticity mechanisms have been associated with engram formation (Josselyn and Tonegawa, 2020). While it has been a matter of thorough debate since the initial reports by Bliss and Lømo (Lømo, 1966; Bliss and Lømo, 1973), today it is generally accepted that LTP is the most likely candidate for a synaptic mechanism underlying learning and memory (reviewed in Malenka, 1994; Stevens, 1998; Lynch, 2004; Dringenberg, 2020). Within this framework, the characterization of mechanisms underlying both LTP and memory will help disentangle the link between both phenomena. In this sense, ERK provides a compelling case study, as there has been extensive research on its role in both LTP and different memory paradigms.

### Extracellular-signal regulated kinase in synaptic plasticity

A large body of work had proposed ERK activation as a key element for LTP and LTD (see Table 1; reviewed in Sweatt, 2004; Thomas and Huganir, 2004; Peng et al., 2010). The first reports of ERK involvement in LTP came from English and Sweatt (1997). The authors showed that using a High Frequency Stimulation (HFS) protocol in the Schaffer Collaterals inputs to CA1 area in rats induced ERK2 phosphorylation and blocking ERK phosphorylation prevented LTP induction. Interestingly, pharmacological blockade had no effect either on the expression of established LTP or short term potentiation (Winder et al., 1999; Mazzucchelli et al., 2002). While these results have been replicated and expanded in rats (English and Sweatt, 1997; Atkins et al., 1998; Selcher et al., 2003), the role of ERK activation in HFS-induced LTP in mice CA1 area is less clear. On one hand, early reports indicated that this kind of LTP was impaired in dominant negative MEK1 (dnMEK1) mice (Kelleher et al., 2004) and was blocked by ERK pharmacological inhibition (Impey et al., 1998). On the other hand, there is also evidence against a role of ERK activity in this process. Winder et al. (1999) first reported an ERK independent form of HFS-induced LTP using a single train of HFS stimulation, and similar results were found using two trains of HFS stimulation (Opazo et al., 2003; Selcher et al., 2003). This stimulation protocol induces a transient form of early LTP, suggesting that ERK is preferentially involved in longer lasting forms of LTP (Huang et al., 2000). In addition, while it was reported that HFS-induced LTP is conserved in ERK1 KO mice (Mazzucchelli et al., 2002) it is important to consider that these mice show enhanced ERK2 signaling, which may rescue the LTP deficient phenotype. These results indicate that when using HFS protocols, ERK involvement may depend on the species and pathways studied, and/or the specific stimulation protocol and experimental conditions used.

**TABLE 1:** Summary of evidence linking ERK to different forms of LTP and LTD.

LTP								
Area	Species	Pathway	Stimulation protocol^a^	ERK activity modulation	ERK activity assessment^b^	Effect on LTP	ERK involvement	References^c^
Hippocampus	Mice	Schaffer Collaterals → CA1	TBS	Pharmachological inhibition, ERK1 KO, Ras-GRF KO, TrkB shc/shc	↑	LTP impaired in ERK1 KO. LTP induction blocked by pharmachological inhibition, no effect on mainteinance.	✓/*X*	**For:** Winder et al., 1999; Mazzucchelli et al., 2002; Selcher et al., 2003; Opazo et al., 2003; Watabe et al., 2000. **Against:** Brambilla et al., 1997; Minichiello et al., 2002
			HFS	Pharmachological inhibition, ERK1 KO, dnMEK1 mutant	↑	Conserved STP, impaired LTP in dnMEK1 mutant. Conserved LTP in ERK1 KO. No effect of pharmachological inhibition or induction blocked by inhibitor, no effect on mainteinance.	✓/*X*	**For:** Impey et al., 1998; Kelleher et al., 2004. **Against:** Mazzucchelli et al., 2002; Selcher et al., 2003; Opazo et al., 2003; Watabe et al., 2000; Winder et al., 1999
	Rat		TBS	Pharmachological inhibition	↑	LTP blocked by ERK inhibitor.	✓	Giovannini et al., 2001
			HFS	Pharmachological inhibition	↑	LTP blocked by ERK inhibitor. No effect on mainteinance.	✓	Atkins et al., 1998; McGahon et al., 1999; Kanterewicz et al., 2000; English and Sweatt, 1997; Selcher et al., 2003
			TEA-LTP	Pharmachological inhibition	–	LTP blocked by ERK inhibitor. No effect on mainteinance.	✓	Kanterewicz et al., 2000
		Mossy Fibers → CA3	HFS	Pharmachological inhibition		Not affected by ERK inhibitor.	*X*	
		Associational/ Commissural Collaterals → CA3	HFS	Pharmachological inhibition		LTP blocked by ERK inhibitor.	✓	
		EC → DG	HFS	Pharmachological inhibition	↑	LTP induction blocked by ERK inhibitor. Mainteinance not affected.	✓	Coogan et al., 1999; Davis et al., 2000
			TEA-LTP	Pharmachological inhibition	–	LTP induction blocked by ERK inhibitor.	✓	Coogan et al., 1999; Davis et al., 2000
Nucleus Accumbens	Mice	Neocortex inputs → Nucleus Accumbens	HFS	ERK1 KO		ERK1 KO present increased ERK2 signaling resulting in enhanced LTP. Pharmacological inhibition of ERK1/2 prevents LTP enhancement.	✓	Mazzucchelli et al., 2002
Perirhinal Cortex		Layer II/III → Layer II	TBS	Ras-GRF1 KO and ERK1 KO		Impaired LTP in Ras-GRF1 KO mice. Enhanced LTP in ERK1 KO mice.	✓	Silingardi et al., 2011
Striatum		Cortico-striatal	TBS	Pharmachological ERK inhibition		Blocked by ERK inhibitor.	✓	Hawes et al., 2013
			HFS	Ras-GRF1 KO		Impaired LTP in Ras-GRF1 KO mice. Enhanced LTP in ERK1 KO mice.	✓	Cerovic et al., 2015
Amygdala	Mice	BLA → LA	TBS	ERK1 KO, Ras-GRF1 KO		No difference between WT and ERK1 KO. Impaired LTP in Ras-GRF1 KO mice.	✓/*X*	**For:** Brambilla et al., 1997. **Against:** Mazzucchelli et al., 2002;
	Rat	MGm/PIN → LA	HFS	Pharmachological ERK inhibition	✓	LTP blocked by ERK inhibitor.	✓	Apergis-Schoute et al., 2005; Ping and Schafe, 2010
		External Capsule → LA			–	LTP blocked by ERK inhibitor. STP not affected.	✓	Huang et al., 2000; Schafe et al., 2000, 2008
		Thalamic afferent fiber → LA				LTP blocked by ERK inhibitor.	✓	

**LTD**								
**Area**	**Species**	**Pathway**	**Stimulation protocol**	**ERK activity modulation**	**ERK activity assessment**	**Effect on LTD**	**ERK involvement**	**References**

Hippocampus	Rat	Schaffer Collaterals → CA1	Muscarinic induced LTD	Pharmachological inhibition	↑	LTD induction but not expression blocked by ERK inhibitor.	✓	Volk et al., 2007; Scheiderer et al., 2008; Mans et al., 2014
			DHPG induced LTD		↑	LTD induction but not expression blocked by ERK inhibitor.	✓	Bolshakov et al., 2000; Gallagher et al., 2004; Chévere-Torres et al., 2012; Potter et al., 2013
			PPS		–	LTD induction blocked by ERK inhibitor.	✓	
			LFS		–	LTD not affected by ERK inhibitor.	*X*	
			Rolipram reinforced LTD		–	LTD induction blocked by ERK inhibitor.	✓	Navakkode et al., 2005
		CA3 → CA1 commisural projection	PPS	Pharmachological inhibition	↑	LTD blocked by ERK inhibitor.	✓	Norman et al., 2000; Thiels et al., 2002
Prefrontal Cortex		Layer I/II to Layer V	Dopamine facilitated HFS-LTD	Pharmachological inhibition	–	LTD blocked by ERK inhibitor.	✓	Otani et al., 1999

^a^While papers have been grouped on the basis of the induction protocols used (i.e. high frequency stimulation, low frequency stimulation), they may not be exactly identical between different groups.

^b^ERK activity was determined in most cases by western blots or immunohistochemistry against phosphorylated ERK. ↑ = Increased phospho-ERK after stimulation. – = ERK activity not determined.

^c^When there is evidence **for** and **against** ERK involvement on LTP, a clear identification of the references is provided.

TBS, theta burst stimulation; HFS, high frequency stimulation; PPS, paired pulse stimulation; TEA-LTP, tetraethylammonium induced LTP; EC, entorhinal cortex; DG, dentate gyrus; DHPG, dihydroxyphenylglycine; PPS, paired pulse stimulation. BLA, basolateral amygdala; LA, lateral amygdala; MGm/PIN, medial geniculate and posterior interlaminar nucleus.

Besides HFS, Theta Burst Stimulation (TBS) has been widely used as LTP inducing stimulus. This kind of stimulation is thought to be more representative of the spontaneous neuronal firing of the hippocampus during behavior (Larson and Lynch, 1986; Larson et al., 1986), and as such, a better model of learning-induced plasticity. Most of the evidence using this kind of stimulation points to the requirement of ERK activity to sustain CA1 LTP in both mice and rats (Table 1). While it has been largely described that LTP is mediated by NMDARs, there is also evidence for the requirement of the BDNF-TrkB pathway activation (Zakharenko et al., 2003; Leal et al., 2014; Panja and Bramham, 2014). However, there is conflicting evidence regarding the requirement of ERK during LTP-induced *via* TrkB receptors dependent on BDNF. Some studies reported it to be ERK-independent (Minichiello et al., 2002; Zakharenko et al., 2003; Minichiello, 2009), whereas there is also evidence of ERK requirement (Ying et al., 2002). Brambilla et al. (1997) also reported that Ras-GRF KO mice have conserved TBS-induced LTP, but as these mice are constitutive GRF knock out, there may be compensatory mechanisms in play that masked the LTP deficient phenotype.

In addition to LTP in the hippocampus, ERK has also been implicated in thalamo-amygdala plasticity. This pathway is of special interest as the thalamus broadcasts auditory information to the amygdala, making it the primary anatomical link between the CS and US in cued fear conditioning (Rogan and LeDoux, 1995; McKernan and Shinnick-Gallagher, 1997; Rogan et al., 1997; Maren, 1999, 2005). It was shown that thalamo-amygdala LTP can be induced *in vivo* in rats *via* stimulation of the MGm/PIN. Moreover, LTP-inducing stimulation increases ERK phosphorylation in the amygdala and thalamus, and both fear conditioning memory and LTP are blocked by infusion of an ERK inhibitor (Apergis-Schoute et al., 2005; Schafe et al., 2008; Ping and Schafe, 2010).

Moreover, ERK has been linked to activity-dependent remodeling of dendritic spines (also known as structural plasticity). ERK activity increases in stimulated spines (Tang and Yasuda, 2017) during structural long-term potentiation and is required for the formation of new dendritic spines following depolarization as well as for AMPAR insertion into synapses from cultured neurons (Wu et al., 2001; Zhu et al., 2002; Goldin and Segal, 2003). Furthermore, increased dendritic spine density upon BDNF treatment in hippocampal pyramidal neurons has shown to be dependent on ERK activation (Alonso et al., 2004) and removal of endogenous BDNF resulted in decreased spine density (Kellner et al., 2014). It has also been shown that BDNF is capable of prolonging the duration of a short lasting LTM from two days to at least seven days, exerting its effect through hippocampal ERK activation (Bekinschtein et al., 2008). This data supports a three-player scheme, encompassing the effects of BDNF on spine morphogenesis, LTM persistence and ERK-dependency.

Evidence regarding ERK scaffold proteins linked to learning and memory is still scarce. Nonetheless, it was described that KSR1-/- mice show deficits in contextual and cued fear conditioning, Morris water maze and passive avoidance as well as theta burst stimulation-induced LTP without altering general behavior (Shalin et al., 2006).

It is not surprising that given the wide variety of experimental protocols and brain areas studied, there is opposing evidence regarding the role of ERK in LTP. It is of particular interest that when using LTP induction protocols that are more closely related to physiological occurring patterns of neuronal activity (Buzsáki, 1989; Hernandez et al., 2005; Larson and Munkácsy, 2015), the majority of the evidence seems to point to a relevant role of ERK. Not only these results suggest that ERK is involved in the establishment of LTP, but also that it plays a role supporting the structural changes that underlie LTP. However, more data addressing this last point is missing and more research is still needed.

### Extracellular-signal regulated kinase in learning and memory

Activation of the ERK pathway has been described in several memory tasks involving different brain regions and animal species (Table 2). ERK activation requirement has been pharmacologically demonstrated in the dorsal hippocampus for Morris water maze (Blum et al., 1999) and in the prefrontal cortex (PFC) for recognition memory (Kelly et al., 2003). The latter was also shown to be partially mediated by dopamine D1 receptors (Nagai et al., 2007). However, ERK activation has also been linked to memory disruption. Adult mice overexpressing the tyrosine phosphatase SHP2 in hippocampus, a model of Noonan syndrome (NS), results in increased baseline excitatory synaptic function and deficits in LTP as well as spatial learning. These deficits can be reversed by a MEK inhibitor, demonstrating that increased basal ERK activity is responsible for the LTP impairments and, consequently, the learning deficits in the mouse model of NS (Lee et al., 2014). Likewise, there is evidence of age-dependent LTM impairment accompanied by overactivation of ERK1 in the medial prefrontal cortex of the triple transgenic mice (3xTg), an animal model of Alzheimer disease (AD) expressing PS1^M146V^, APP_Swe_, and tau^P301L^ transgenes (Oddo et al., 2003), in which local ERK inhibition rescues recognition memory deficits (Feld et al., 2014). Thus, in both models excessive increase of ERK activity explains cognitive deficit, and inhibition of overactivation was enough to restore normal LTM, supporting the need for fine-tuning of this pathway in mnesic processes.

**TABLE 2:** Outline of research associating ERK activity to different forms of learning and memory.

Species	Behavioral task	Expeimental manipulation	Effect on ERK activity	Behavioral outcome	References
*Aplysia*	Long Term Facilitation	Pharmachological inhibition	↑	LTF blocked by MAPK inhibition. No effect on STF.	Martin et al., 1997; Purcell et al., 2003
*Hermissenda*	Classical Conditioning (Foot length retraction)	–	↑	–	Crow et al., 1998
*Lymnaea*	Food reward conditioning	Pharmachological inhibition	↑	MAPK inhibition blocks memory formation.	Ribeiro et al., 2005
*Drosophila*	Olfactory Aversive Conditioning	Pharmachological inhibition	↑	Pharmachological inhibition blocks LTM. ERK determines effective trial spacing for LTM induction.	Pagani et al., 2009; Li et al., 2016; Miyashita et al., 2018; Zhang et al., 2018; Awata et al., 2019
*Neohelice*	Context-Signal Learning	Pharmachological inhibition	↑	Pharmachological inhibition blocks memory formation.	Feld et al., 2005
	Classical Conditioning				Ojea Ramos et al., 2021
Mice/Rat	Fear Conditioning	Pharmachological inhibition, ERK2 KO, dnMEK, RasGRF2 KO	↑	ERK inhibition in the hippocampus blocks LTM consolidation. ERK inhibition in the Amygdala blocks FC extinction and Reconsolidation. ERK2 KO, dnMEK and RasGRF2 KO mice have impired LTM.	Atkins et al., 1998; Selcher et al., 1999; Kelleher et al., 2004; Duvarci et al., 2005; Herry et al., 2006; Trifilieff et al., 2006, 2007; Satoh et al., 2007; Guedea et al., 2011; Besnard et al., 2013, 2014; Zamorano et al., 2018
	Morris Water Maze	Pharmachological inhibition, ERK2 KO, dnMEK	↑	ERK inhibition in the hippocampus or EC block LTM. ERK2 KO and dnMEK mice have impaired LTM.	Blum et al., 1999; Selcher et al., 1999; Hebert and Dash, 2002; Kelleher et al., 2004; Satoh et al., 2007
	Cocaine/Morphine Induced Conditioned Place Preference	Pharmachological inhibition	↑	ERK inhibition impaired reconsolidation and LTM.	Miller and Marshall, 2005; Valjent et al., 2000, 2006; Lin et al., 2010; Li et al., 2011; Papale et al., 2016
	Inhibitory Avoidance	Pharmachological inhibition	↑	EKR inhibition impaired LTM, retrieval an memory reconsolidation.	Walz et al., 1999; Cammarota et al., 2000; Kim et al., 2012; Krawczyk et al., 2015; Fukushima et al., 2021
	Object Recognition	Pharmachological inhibition, ERK2 KO	↑	ERK inhibition impaired memory consolidation and reconsolidation.	Kelly et al., 2003; Fernandez et al., 2008; Vithayathil et al., 2017

LTF, long term facilitation; STF, short term facilitation; LTM, long term memory; FC, fear conditioning; EC, entorhinal cortex.

The activation of ERK by drugs of abuse in brain regions related to reward (Table 2) is necessary for the induction of immediate early genes and depends on dopamine D1 and glutamate receptors. Blocking ERK prevents changes in behavior including acquisition of a conditioned locomotor response triggered by a cocaine- or D-amphetamine-paired context and conditioned place preference (Girault et al., 2007). In addition, nicotine administration enhances contextual fear conditioning acquisition by ERK activation (Raybuck and Gould, 2007). Moreover, pharmacological activation of βARs in the LA resulted in increased freezing after a weak cued-fear conditioning training protocol in rats in which ERK activation was essential for consolidating the learned association (Schiff et al., 2017). Furthermore, sertraline, a selective serotonin reuptake inhibitor (SSRI) that stimulates synaptic plasticity and neurogenesis, significantly improved spatial memory learning in both young and old mice. The most likely mechanism underlying this effect is by the activation of serotonin (5-HT) receptors that induce ERK activation, up-regulation of brain BDNF and Bcl-2 (Taler et al., 2013).

In addition, ERK phosphorylation is also necessary for memory in invertebrates (Table 2). Examples include LTM in *Aplysia* 5-HT-mediated sensitization of the siphon retraction reflex (Martin et al., 1997; Philips et al., 2013), in *Drosophila* olfactory conditioning (Pagani et al., 2009; Miyashita et al., 2018), in *Lymnaea* food-reward conditioning (Ribeiro et al., 2005); in *Hermissenda* classical conditioning of foot retraction (Crow et al., 1998) and in associative fear learning in *Neohelice* (Feld et al., 2005; Ojea Ramos et al., 2021).

The group of Josselyn has proposed that neurons overexpressing CREB are preferentially allocated to the fear memory trace due to its increasing excitability function (Yiu et al., 2014), in part by decreasing voltage-gated potassium channels in the amygdala and the hippocampus (Viosca et al., 2009). However, since in these experiments CREB is overexpressed by viral injection, there is no information about the time course of the endogenous CREB expression. Likewise, ERK may also contribute to increasing neuronal excitability and thus neuronal recruitment to the engram, not only by mediating CREB activation *via* MSK and RSK2 (Hauge and Frödin, 2006; Sindreu et al., 2007), but also by direct phosphorylation of Kv4.2 channels decreasing potassium current in hippocampal CA1 neurons (Adams et al., 2000; Schrader et al., 2006) and in dendrites by PKA and PKC pathways converging on ERK (Yuan et al., 2002). However, since no studies have directly addressed this question, it is still unknown whether ERK activation may lead to neuronal allocation to the engram.

It has been established that ERK is also relevant for memory processes taking place after the initial consolidation has occurred. The presentation of a long continuous or several discrete unreinforced reminders leads to extinction, a process that entails the consolidation of a new memory and inhibition of the original one (Pavlov, 1927; Bouton, 2004; Hermans et al., 2006). In contrast, the presentation of few unreinforced reminders lead to memory reconsolidation, triggering an initial destabilization and posterior re-stabilization of the memory trace, thus allowing for modifications such as strengthening, update or even erasure (Nader et al., 2000; Sara, 2000; Pedreira et al., 2004). Both reconsolidation and extinction require activation of ERK (Duvarci et al., 2005; Herry et al., 2006), although in some cases, it has also been observed that the avoidance memory reactivation process induces a negative regulation of ERK in the amygdala, prefrontal cortex (involved in emotional evocation) (Botreau and Gisquet-Verrier, 2006) and hippocampus (Krawczyk et al., 2015, 2016). Many efforts are focused on understanding the role of ERK into these processes. Interestingly, since whether the triggered process is reconsolidation or extinction only depends on the accumulation of time spent under non-reinforced reminder presentation, the study of ERK may help to elucidate the mechanisms underlying the transition between these two processes (Merlo et al., 2018; Fukushima et al., 2021). However, we will not delve on this topic since it exceeds the scope of this review (for the role of ERK in reconsolidation and extinction see Cestari et al., 2014; Medina and Viola, 2018; Krawczyk et al., 2019).

Taken together, this evidence shows that ERK inhibition impairs memory formation in multiple tasks in different species, and that overactivation leads to memory deficits which can be prevented by ERK downregulation, strongly suggesting that ERK activation levels critically contribute to memory trace formation.

### Differential role of ERK1 and ERK2 in memory formation?

The emergence of ERK1 and ERK2 isoforms has been explained as a consequence of a whole genome duplication event early in the evolution of the vertebrate phylum (Buscà et al., 2015). Their primary structures are 84% identical across mammals (Eblen, 2018) although ERK1 protein is larger than ERK2 mainly due to a larger N-terminus, and ERK2 is expressed at higher levels than ERK1 in most mammalian tissues.

A thorough review of published studies on the role of ERK1 vs. ERK2 has largely favored the functional redundancy hypothesis against isoform specificity (Buscà et al., 2015). However, while ERK1 null mice are viable and fertile (Selcher et al., 2001; Mazzucchelli et al., 2002), ERK2 constitutive knockouts are embryonic lethal (Lefloch et al., 2008; Satoh et al., 2011). Results investigating LTM in ERK1 KO mice are controversial. Findings showed no effect on acquisition or long-term retention of either contextual/cue fear conditioning or passive avoidance memory and hippocampal high frequency stimulation (HFS) induced CA1 LTP (Selcher et al., 2001), whereas others found improvement in active and passive avoidance memory and theta burst induced LTP (Mazzucchelli et al., 2002; Table 1). Differential ERK1/ERK2 regional distribution in rat brain (Ortiz et al., 1995) also suggests a possible regulation of isoform function. Moreover, several reports have shown unexpected interplay between isoforms pointing to specific roles for ERK1 and ERK2 at least in plasticity and memory.

Moreover, mice lacking ERK1 presented a dramatic enhancement of striatum-dependent long-term memory, correlating with a facilitation of long-term potentiation in the nucleus accumbens and stimulus-dependent increased ERK2 signaling, suggesting a regulatory action of one isoform onto the other (Mazzucchelli et al., 2002). Interestingly, later studies also showed that ERK1 KO mice had increased ERK2 activity, as well as enhanced LTP and LTD in perirhinal cortex (PRHC), a brain area known to play an essential role in familiarity-based object recognition memory. These animals exhibited better long-lasting recognition memory compared to *wild-type* mice (Silingardi et al., 2011). Although these findings might seem puzzling, attention must be paid to the fact that not only this pathway is being considered in the context of plasticity, learning and memory, but it has also a profound effect on nervous system development and consequently, it is not possible to conclude independently of the temporal point of the manipulations performed (Vithayathil et al., 2017). Finally, functional differences between both isoforms, have been attributed to the fact that ERK cytoplasmic-nuclear trafficking depends on their N-terminus, accounting for the reduced nuclear shuttling rate of ERK1 compared to ERK2, and consequently ERK1 reduced capability to carry proliferative signals to the nucleus (Marchi et al., 2008).

In spite of this evidence, it is still a matter of debate whether ERK1 and ERK2 are equally relevant for learning and memory processes.

### Temporal integration of extracellular-signal regulated kinase during memory formation

Spacing effect is a major phenomenon occurring during learning which has been characterized in different experimental memory models, in both invertebrates (Philips et al., 2007; Pagani et al., 2009; Ojea Ramos et al., 2021), and vertebrates (Bello-Medina et al., 2013; Aziz et al., 2014; Pandey et al., 2015), including humans (Ebbinghaus, 1885; Vlach et al., 2008). It refers to the greater effectiveness of training protocols where trials are spaced in time, compared to those in which trials are presented in a continuous fashion (without or with brief inter-trial intervals, ITI). However, this general rule is difficult to interpret when comparing learning tasks used in vertebrates and invertebrates. One hypothesis to explain this effect assumes that there is a refractory period in learning during which the second of two stimuli is ineffective to improve the outcome of the first. Therefore, including a prolonged ITI during training, would allow for this refractory period to be overcome. Alternatively, the first trial of a spaced training would have a “priming” effect on the synapses, so that the molecular processes that occur toward the end of training are reinforced enabling LTM formation (Smolen et al., 2016). Moreover, it has been posited that the net balance between CREB activators and repressors increases after training, favoring activators and thus, shifting the outcome toward maximal LTM formation at longer ITIs (Yin et al., 1995). Nevertheless, these hypotheses are not mutually exclusive.

One example is the well-known sensitization learning of the *Aplysia* mollusk siphon retraction reflex. While four training trials presented without an ITI are not capable of generating a LTM, it is enough if they are separated by a 15 min ITI (Philips et al., 2007). Moreover, presenting only the first and last trials (two-trial, 45 min-ITI training), which maintains the total duration of the session, also induced LTM (Philips et al., 2013). The success of this protocol was shown to be due to a delayed protein synthesis-dependent nuclear MAPK activity that established a unique molecular context. Similar results were obtained using the semi-terrestrial crab *Neohelice granulata*. In this species, a standard visual stimulation protocol (15 trials, 3 min-ITI) induces a delayed peak of ERK activity (1 h) after training (Feld et al., 2005), while the two-trial protocol (45 min-ITI) reduces the activation time to 5 min (Ojea Ramos et al., 2021). In both species, inter-trial ERK inhibition impaired LTM, highlighting the relevance of either the total duration of the stimulation protocol or the length of the ITI in order to induce effective ERK activation.

Experiments in the fruit fly *Drosophila melanogaster* demonstrated that protein tyrosine phosphatase SHP2 (corkscrew) altered Ras/ERK pathway activation waves and shortened ITIs required for LTM formation (Pagani et al., 2009). In this work, ERK phosphorylation took place during ITI and trial presentation canceled this activation, thus longer ITI allowed for prolonged ERK kinetics. Similar findings, although measured at different time points, were reported by Miyashita et al. (2018). In this study, the authors showed that ERK activity increases during ITI in spaced training, inducing ERK/CREB/c-Fos cycling, which defines potential engram cells. Furthermore, disruption of *Drosophila* D1 dopamine receptors, and Ca^2 +^/calmodulin regulated adenylyl cyclase (AC), prevented increases in pERK and subsequent c-Fos/CREB cycling (Miyashita et al., 2018). Supporting previous findings, Awata and coworkers also found that distinct parallel circuits in the mushroom bodies subserves, through pERK expression, spacing effect sparse coding information *via* dopamine signaling and memory consolidation (Awata et al., 2019). Interestingly, the authors also observed differential threshold activation in neuronal subtypes, suggesting that neuronal activity *per se* is not sufficient to induce activation of the pathway. Noteworthy, PP1 or CaNB2 loss of function in these flies is sufficient to bypass the requirements for ITI during training but pERK still needs to be activated for a sufficient amount of time to allow c-Fos/CREB cycling to occur (Miyashita et al., 2018). Likewise, it has been largely demonstrated in *Aplysia* that the MEK/ERK pathway contributes to 5-HT-induced phosphorylation of CREB1 *via* RSK or PKA, as well as LTF (Sharma and Carew, 2004). Recent studies combining experimental and computational approaches propose positive feedforward and negative feedback loops leading to different ERK activation kinetics, revealing the importance of signaling pathways’ fine-tuning (Liu et al., 2020; Zhang et al., 2021). Although, to our knowledge there is no data supporting direct phosphorylation of CREB2 by ERK, this potential interaction may relieve the repression exerted by the repressor, inducing gene expression (Abel et al., 1998; Fioravante et al., 2006). Taken together, these results in invertebrate memory studies highlight a central role of ERK activation and inhibition periods during this process.

Both vertebrates and invertebrates seem to be capable of memory enhancement after spaced training although a reduced number of trials are delivered. Rats under massed fear conditioning training show no or weak LTM compared with rats given the same amount of light–shock pairings presented in a spaced manner (Josselyn et al., 2001). In addition, two sessions of weak spatial object recognition (SOR) task, each of which does not induce LTM independently, elicited 24h retention when delivered in a spaced fashion. Memory enhancement by spaced training was dependent on ERK activation in the dorsal hippocampus and open field exploration rescued SOR memory impairment induced by ERK inhibition (Tintorelli et al., 2020). According to the authors, these observations could be interpreted under the behavioral tagging (BT) hypothesis that explains how a weak event that induces transient changes in the brain can establish long-lasting phenomena through a tagging and capture process achieving synaptic specificity and persistence of experience-induced plastic changes (Viola et al., 2014).

Temporally spaced synaptic stimulation in slices and behavioral training improved synaptic potentiation and long-term memory for contextual fear conditioning in mice, respectively (Scharf et al., 2002). Moreover, stimulation of the hippocampal CA1 with successive bouts of theta bursts, which are considered a more physiological frequency, enhanced previously saturated LTP only when spaced by long intervals (e.g. 1h or longer). This enhancement may be due to recruitment of synapses that were “missed” by the first stimulation bout (Kramár et al., 2012). In cultured hippocampal neurons, spaced but not massed depolarizations evoke persistent activation of ERK, critical for protrusion of new dendritic filopodia that also remained stable for hours (Wu et al., 2001). In addition, ERK activity in the amygdala increased one hour after a first fear-training session but not after a second one (Parsons and Davis, 2012) albeit activation at earlier times after the second trial should not be discarded (Ojea Ramos et al., 2021). Furthermore, dorsal hippocampal synaptic ERK activation induced after spaced short trials of an object-location task was associated with LTM formation in Fmr1 KO mice model of fragile X syndrome (Seese et al., 2014).

Thus, spacing effect has been reported in a plethora of studies involving different phenomena including different forms of plasticity, learning and memory. However, whether it mechanistically relies on the same targets in vertebrates and invertebrates has not been fully ascertained. LTM induction after spaced training in flies was shown to depend on relative amounts of CREB activators and repressors (Yin et al., 1995), while in mice lacking the alpha and delta isoforms of CREB, spaced training selectively rescues long-term memory (Kogan et al., 1997).

Taken together, these findings demonstrate that the spacing effect allows for enhanced LTM expression and for different learned experiences to be temporally integrated in an ERK-dependent fashion. ERK activation (and inhibition) kinetics outlines the effectiveness of ITI duration for a successful LTM formation. In this sense, the first trial triggers a loop of kinases, transcription factors and immediate early genes (e.g. ERK/CREB/c-Fos) with a certain time course that allows signal integration with other molecular events. In this regard, while a premature second trial presentation would impair this loop to continue, preventing LTM formation, a prolonged ITI would allow for this cycle to be fulfilled, inducing LTM formation. This mechanism could be then integrated among different circuits enabling memory formation across different areas and for longer periods (e.g. systems consolidation).

### Extracellular-signal regulated kinase kinetics in aversive memories

A large body of work has drawn particular attention to the role of ERK in aversive memories. The two most extended tasks performed in these studies are inhibitory avoidance and pavlovian fear conditioning.

In the inhibitory avoidance (IA) task animals learn to avoid an aversive stimulus (e.g. a foot-shock) by inhibiting a response of locomotion and exploration. Thus, to withhold stepping through a hole into a dark compartment (“step through” version), or stepping down from a platform (“step down” version). For the purpose of this review, inhibitory avoidance encompasses step-down and step-through versions. As a result of acquisition, animals increase the latency to step into the compartment where they received the shock.

In the Pavlovian cued fear conditioning (FC), a neutral tone (conditioned stimulus, CS) is paired with an aversive foot-shock (unconditioned stimulus, US) (paired conditioning). Since in this case the context is also associated (context in background) to the US, the tone test is performed in a different environment. In another variant of the task, there is a lack of contingency between the discrete CS (tone) and the US (unpaired conditioning), which favors the association with the context (context in foreground). In both cases, once the association is formed, the presentation of the tone or the context respectively, elicits freezing as the conditioned response. Moreover, both types of conditioning induce fear to the context, but they result in distinct contextual processing that depend on the amygdala and hippocampus (Kim and Fanselow, 1992; Phillips and LeDoux, 1994; Desmedt et al., 1998, 2003; Calandreau et al., 2005).

**FIGURE 2:**
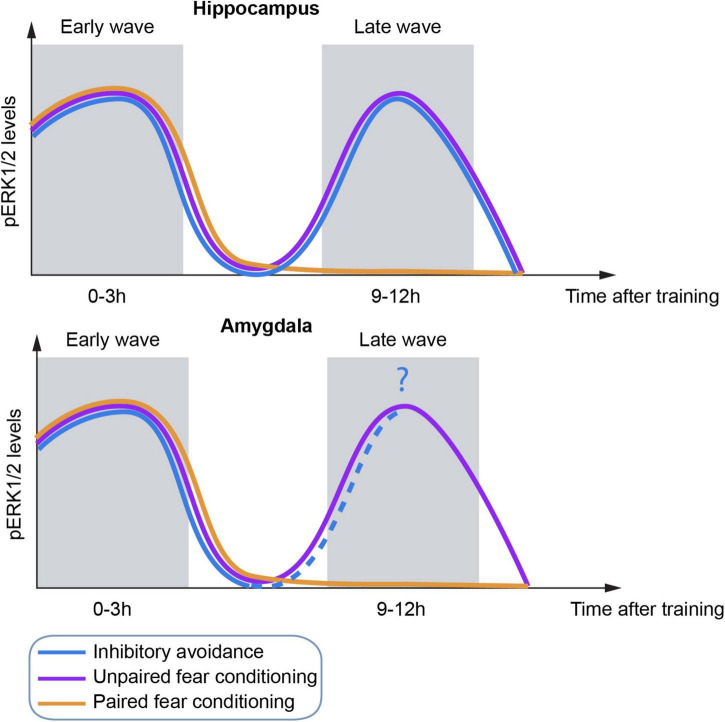
Schematic representation of temporal dynamics of ERK activation in the hippocampus **(top)** and amygdala **(bottom)** by three different fear behavioral tasks.

Although IA and FC are very different paradigms, they share interesting similarities regarding the activation kinetics of ERK (Figure 2). Several studies have shown an increase of ERK phosphorylation in both hippocampus and amygdala (mainly LA) at early times (0–3 h) after acquisition of inhibitory avoidance (Alonso et al., 2002; Cammarota et al., 2008) and both FC protocols (Atkins et al., 1998; Schafe et al., 2000; Trifilieff et al., 2006, 2007; Besnard et al., 2014). Interestingly, IA and unpaired, but not paired, FC triggered a second wave of ERK activation at later times (10–12 h) after training (Trifilieff et al., 2006, 2007; Bekinschtein et al., 2008). As anticipated, CREB activation also followed ERK kinetics in both FC protocols (Trifilieff et al., 2006). Foundational work by Grecksch and Matthies (Grecksch and Matthies, 1980) as well as others, supported that two protein synthesis waves are necessary for memory consolidation, positing the requirement of the first wave in order to allow the second one to occur. In this sense, a second wave was also observed for the IEG c-Fos (Katche et al., 2010) and BDNF (Alonso et al., 2002, 2004; Bekinschtein et al., 2008) mostly related to memory persistence.

Strikingly, although all three protocols induce the first wave of activation of ERK at a similar time point, the second wave was not dependent on the occurrence of the first one, at least for unpaired FC (Trifilieff et al., 2006; Figure 2). Importantly, ERK activation is required for consolidation of these tasks since inhibition of any ERK wave resulted in memory impairment (Atkins et al., 1998; Walz et al., 1999, 2000; Schafe et al., 2000; Trifilieff et al., 2006).

One possible explanation may be that ERK functions as a coincidence detector, where the afferents containing the auditory or action (step-down/step-through) inputs followed by the shock information (Nabavi et al., 2014; Tovote et al., 2015) coincide in the amygdala and the hippocampus triggering the first wave of ERK activation, although this could also be due to a non-associative sensory activation (Alonso et al., 2002).

In contrast, the second wave of ERK activation was only present in unpaired FC and strong IA. Since the saliency of the context in these two protocols is greater than in paired FC, it makes sense that they share the underlying molecular principles. If the second wave of ERK activation is independent of the first one, what triggers ERK activation in the absence of stimuli? One hypothesis could come from hippocampal place cells, neurons that fire preferentially at specific locations within a spatial environment (O’Keefe and Dostrovsky, 1971). On the one hand, there is more remapping during unpaired FC than during paired FC (Moita et al., 2004), which would explain the difference in the ERK kinetics between both FC protocols. On the other hand, place cells are able to replay the hippocampal representation of the environment in the absence of stimuli, supporting their role in memory consolidation (Wilson and McNaughton, 1994; Jackson et al., 2006; Carr et al., 2011), which happens during sharp-wave/ripple (SPWs) events (Buzsaki et al., 1992). Moreover, there is evidence of replay of IA occurring during the inhibitory action at retrieval and without exploring the actual feared zone (Wu et al., 2017). Furthermore, SPWs facilitate the strengthening of memories (Dupret et al., 2010), strongly indicative of a role of the second ERK wave in memory persistence (Bekinschtein et al., 2008; Miyashita et al., 2008). Likewise, the interconnectivity between the hippocampus and the amygdala would allow for the transmission of information across these two areas and therefore the observed ERK activation at similar times (Tovote et al., 2015).

Although the hypothesis may be plausible, so far there is no direct evidence that supports this and further experiments should be considered (see section “Conclusions and perspectives”). Another unexplored aspect of the two-wave ERK phosphorylation is whether the activation that occurs in the second wave is in the same neurons compared to the first one or in a subset of them, similar to what was observed for the IEG ARC (activity-regulated cytoskeleton-associated protein) activation in a spatial maze (Ramirez-Amaya et al., 2005).

The similar kinetics observed in different fear memory protocols together with the evidence that increased and decreased ERK activity influences the ability of LTM to be formed, suggest a specific role of ERK activation function during memory formation. Moreover, the relevance of ERK activation during temporal integration argues in favor of a distinct participation of the kinase in the memory trace, rather than a general activity marker.

## Conclusions and perspectives

Along this review we have revised data on the activation of ERK in neurons, ranging from signals that trigger the pathway to the subcellular targets underlying learning-related plasticity. The heterogeneity of neurotransmitter signals triggering ERK phosphorylation may account for a general role of this kinase in memory plasticity. Though the requirement of enhanced ERK activation by multiple systems for memory formation remains elusive, it suggests an integrative function of the kinase. This computation would allow for various stimuli converging at the single neuron level to modulate ERK activation dynamics according to their specific pattern of occurrence, possibly allowing these neurons to be recruited into the engram.

Likewise, there is a wide variety of ERK actions including binding to actin filaments and local translation initiation in dendrites, suggesting a role in stabilizing structural changes in dendritic spines. In turn, these changes may lead to the maintenance and strengthening of certain synapsis that may be fundamental for LTM.

The transcription factor CREB is able to increase neuronal excitability which results in the recruitment of neurons to the engram (Yiu et al., 2014). ERK activation of transcription factors including CREB, as well as facilitating transcription by crosstalk with HATs reveals a tight association between ERK effects on gene expression regulation and memory formation. Together with the ability of blocking potassium channels, thus increasing neuronal excitability *per se*, this evidence suggests a role of ERK not only in synaptic plasticity necessary for memory formation, but also in the engagement of neurons into the memory trace. Although it remains an open question whether increasing ERK activity in certain conditions might also increase particular neurons’ probability to be included in a particular memory engram, it was recently reported (Zamorano et al., 2018) that ERK is preferentially re-activated during memory retrieval in the same neurons that were activated during acquisition, underpinning a first step to determining whether ERK is a viable ‘engram marker’.

The concerted activation of ERK at similar times by different memory tasks and in various brain regions might suggest that ERK is required in a brain-wide circuit-specific neuronal activation fashion. Moreover, the temporal integration of ERK activation during memory formation reveals an overlap between parallel mechanisms associated with memory. Depending on temporal constraints and the specific elements involved, these shared processes may either interfere with each other resulting in memory impairment or allow for a synergistic effect and subsequently, memory enhancement.

Although ERK activation kinetics may reflect neuronal circuit activity relevant for learning, the direct link between these two phenomena is still missing. Moreover, what does an increase in ERK activation mean? More neurons in which ERK is getting activated or more activation at the level of each single neuron? Thanks to new technological approaches that simultaneously record molecular activation by FRET biosensors together with neuronal activity with calcium imaging (Laviv et al., 2020), it might now be possible to address this type of question.

Evidence involved ERK dysregulation as a contributing factor to memory deficits observed in brain disorders. There remains, however, some outstanding gaps in our understanding to be filled and some difficult issues to be resolved. Overactivation of the ERK pathway may explain some of the findings reported in AD models, in particular, the fact that ERK inhibition rescues memory deficits. In contrast, in most of the learning tasks in healthy animals, inhibition of ERK resulted in memory impairment, indicating the importance of ERK activation homeostasis for memory stabilization.

All together, this evidence indicates that ERK may function as a molecular hub orchestrating neuronal plasticity, contributing to memory trace recruitment, and therefore, a key target for therapies for several brain disorders.

## Author contributions

MF and MSF designed and wrote the manuscript with equal contribution. MSF made the artwork. SOR designed and constructed the tables and contributed to the writing of the manuscript.
